# Responses of Fish Zeta Diversity (ζ) to Human Pressure and Cumulative Effects: A Feasibility Study of Fishing Ban Measures in the Pearl River Basin, China

**DOI:** 10.3390/biology14070796

**Published:** 2025-06-30

**Authors:** Jiayang He, Hao Liu, Xianda Bi, Zhiqiang Wu

**Affiliations:** 1 Guangxi Academy of Fishery Sciences, Ministry of Agriculture and Rural Affairs, China (Guangxi)-ASEAN Key Laboratory of Comprehensive Exploitation and Utilization of Aquatic Germplasm Resources, Nanning 530021, China; hejiayang@ihb.ac.cn; 2 Institute of Hydrobiology, Chinese Academy of Sciences, Wuhan 430072, China; 3 College of Environmental Science and Engineering, Guilin University of Technology, Guilin 541004, China; liuhaoixx@163.com

**Keywords:** zeta diversity, beta diversity, cumulative effects, freshwater fish, human pressures, the Pearl River Basin, fishing ban measures

## Abstract

Amid declining fish diversity and human pressures in freshwater ecosystems, robust basin-scale assessments are vital for effective fisheries management. This study collated nearly four decades of fishery yields from the Pearl and Yangtze Rivers to identify conservation priorities in the Pearl River Basin. It introduced a novel cumulative effect indicator based on zeta diversity—a biodiversity pattern metric—integrated with cumulative effects analysis for management decision-making. The research employed a multi-site generalized dissimilarity model to examine the non-linear relationships between fish species composition (ζ_n_) and human pressures, environmental factors, and geospatial variations across elevation gradients. The cumulative effect indicator, reflecting responses to anthropogenic stress when assessing ζ_2_ (related to β diversity), helped evaluate basins for conservation or restoration needs based on their unique or homogenized biotic communities. The results suggest that ζ diversity in low-elevation sub-basins has a stronger filtering effect on ζ by human pressures than in mid- to high-elevation sub-basins, where community aggregation is more random. The impact varied with diversity aspects (nestedness vs. turnover) and zeta order. A negative correlation between cumulative effects and community uniqueness validated the novel cumulative effect indicator’s effectiveness for guiding restoration in the Pearl River Delta, potential fishing bans, and karst conservation. This approach offers a theoretical basis for prioritizing areas for freshwater fish diversity conservation and fishing restrictions in the Pearl River Basin.

## Simple Summary

This study analyzed nearly four decades of fish data from China’s Pearl and Yangtze Rivers to develop a new method for identifying areas that need conservation or restoration due to declining freshwater fish diversity. Researchers created a novel indicator that measures the combined impacts of human activities (fishing, agriculture, and urban development) on fish communities by examining how different species are distributed across the river basin and respond to various pressures. They found that fish communities in low-elevation areas are more severely affected by human activities than those in mountainous regions, with some areas maintaining unique fish communities that require immediate protection while others have become homogenized and need restoration efforts. This research provides a scientific framework for decision-makers to prioritize conservation areas, implement fishing restrictions where needed, and guide restoration efforts in degraded regions, offering an approach that can be applied to other river systems worldwide facing similar challenges from human development and declining fish populations.

## 1. Introduction

Fish biodiversity is fundamental to human well-being [1]. More than half of the global GDP depends on the natural environment, undertaking the risk of disruption and devaluation [2]. Of these, fisheries, as a typical representative, cannot be neglected due to their contribution to global economic development [3]. Anthropogenic biodiversity decline has become a sequential focus of government and the public. Many of the multiple major drivers of fish species extinction and ecosystem dysfunction have become more prominent alongside economic development [4]. Fortunately, awareness of the conservation of natural systems and participation in conservation activities have been strengthened in past decades [5,6]. Notable initiatives include the establishment of over 18,000 marine protected areas globally, covering approximately 8% of ocean areas, the implementation of sustainable fisheries certification programs, such as MSC, which has certified over 400 fisheries worldwide, and international frameworks like the Convention on Biological Diversity that has mobilized conservation actions across 196 countries with measurable biodiversity targets. Indisputably, however, there is a massive divide between people’s expectations for action to ameliorate fish biodiversity decline and the decision-making framework in the world, especially in developing countries [2,7].

Compared to developed countries, maintaining a balance between economic development and ecological protection is extremely difficult in developing countries when compensation and employment subsidies are absent [8,9,10,11,12,13]. Fishing management, even as a widely accepted policy worldwide, has triggered conflicts between ecological and economic performance in underdeveloped areas. Following the fishing management law, fishermen could not obtain alternative incomes [14,15]. The Pearl River Basin, similar to the Yangtze River Basin, has experienced extremely heavy perturbations and human stress from anthropogenic activities during the past decades as a consequence of the development of the regional economy [16]. These anthropogenic stressors include intensive industrial development (particularly manufacturing and petrochemical industries, contributing >60% of regional GDP), rapid urbanization, with population density exceeding 500 people/km^2^ in delta regions, extensive infrastructure construction, including dam building (over 100 large dams constructed since 1980s), agricultural intensification, with 45% land conversion to cultivated areas, and sand mining activities that have extracted >1.5 billion tons annually, collectively contributing to habitat fragmentation, water quality degradation, and species displacement, with fishing pressure accounting for approximately 15–20% of the total biodiversity impact. Therefore, the Pearl River Basin, one of China’s significantly and quickly developing economic zones, provides the possibility to apply fishing management or bans for sustainable development of fisheries and conservation of fish biodiversity.

The 10-year fishing ban was implemented in the Yangtze River Basin in 2021 [17]. Meanwhile, the Yangtze River Protection Law was enacted in 2021 to restore the river further [18]. These are crucial for the government and the public in protecting fish biodiversity and environmental protection, and a concession of economic development to natural restoration. Notably, as an excellent fish biodiversity basin in China, sustainable fisheries and fish biodiversity security in the Pearl River Basin have undertaken the sustained stress from overfishing and anthropogenic perturbations [7,19]. Owing to the lack of effective fisheries management policies and enforcement measures, the fishing ban policy in the Pearl River Basin remains insufficient and stagnant. One of the most important reasons for the underdeveloped policy is that it is not supported by adequate scientific investigation. The potential policies and Pearl River Protection law being proposed by the Chinese government would be the first significant step to achieving major sustainability objectives of target ten of the Kunming-Montreal Global Biodiversity Framework and the goal of the Sustainable Development Goals (SDGs) of the United Nations [20,21,22,23].

To address these challenges, several analytical approaches are essential for assessing biodiversity changes and informing conservation priorities in river basins. Beta diversity assessment, particularly through Local Contributions to Beta Diversity (LCBD), provides crucial metrics for identifying sites with unique species compositions that require conservation attention [24,25]. Sites with high LCBD values indicate either unique ecological conditions worth preserving or degraded areas experiencing distinct species loss patterns, thus serving as early warning indicators for ecosystem health decline [24]. For instance, a river reach with elevated LCBD may signal localized pollution impacts or habitat fragmentation that has altered its aquatic communities’ assemblage compared to neighboring sites [26,27]. Advanced methods, such as multi-site generalized dissimilarity models (MS-GDMs), enhance our understanding of how environmental and human pressures influence species turnover across various sites by utilizing zeta diversity (ζ_n_) metrics [28,29]. MS-GDM provides a robust theoretical framework for spatial inference by capturing non-linear responses of species compositional turnover to environmental gradients, while zeta diversity quantifies the shared species patterns across multiple sites simultaneously, making their combination particularly suitable for identifying biodiversity hotspots and cumulative impact zones across heterogeneous landscapes. This synergy enables spatially explicit conservation prioritization by linking mechanistic understanding of species responses with multi-site community patterns [27].

These approaches help distinguish between the impacts on common species that support ecosystem functions and rare species that face greater extinction risks. Recent international studies have demonstrated the effectiveness of zeta diversity in watershed conservation prioritization, from identifying critical wetland complexes that promote biodiversity [30] to revealing metacommunity assembly processes in freshwater systems through functional zeta diversity [31]. Additionally, cumulative effect assessments that quantify the combined impact of multiple human pressures are increasingly vital for effective ecosystem management [32,33]. Unlike traditional indices (α or β diversity) that focus on single-site patterns or pairwise comparisons, our cumulative effect indicator uniquely integrates non-linear responses of species turnover to multiple stressors through I-spline transformations, enabling managers to identify areas requiring conservation versus restoration based on their vulnerability to anthropogenic impacts [27]. By integrating MS-GDMs with LCBD, we can develop metrics based on species turnover that offer novel perspectives for managing biodiversity under multiple stressors [27,34,35].

In this study, nearly 40 years of freshwater fisheries production in the Pearl River are collected, fish species turnover is assessed by MS-GDM in response to human pressures and environmental and geospatial differences between sub-basins, and cumulative effect indicators are developed based on the sum of responses to human pressure variables. Therefore, the main aims of this study are to (1) investigate the situation of fishery productively during the past 40 years and to measure the difference in productivity between the Pearl River and Yangtze River Basin, (2) focus on the impacts of different human pressures on species compositional turnover in the Pearl River Basin through the quantification of fish community responses, and (3) compare LCBD of the Pearl River Basin and cumulative effect indicators, combining with a non-linear response, to determine the correlation between a particular species composition and cumulative effect. The present study systematically gathers data on fisheries economics and fish biodiversity in the Pearl River Basin over the past 39 years, which provides a better understanding of the practical and theoretical basis for fisheries’ resilient and sustainable development.

## 2. Methods

### 2.1. Study Area

The Pearl River Basin is located at 102°15′~115°35′ E, 21°50′~26°48′ N, with an area of 45.4 × 10^4^ km^2^, annual average temperature of 14 °C~22 °C, annual average precipitation of 1200 mm~2000 mm, and annual average runoff of 0.338 × 10^12^ m^3^. The annual runoff is more than 3.49 × 10^12^ m^3^, ranking second in China’s river system [36]. The unique natural conditions of the Pearl River Basin (karst landform), including extensive underground river systems, varied water chemistry with high calcium carbonate content, diverse microhabitats, such as caves and sinkholes, and fragmented hydrological connectivity that creates isolated aquatic environments favoring speciation and endemism, breed a wealth of freshwater fish whose diversity, endemic species, and endangered species rank first in China [19]. Since 2017, the fishing ban has been extended in the Pearl River Basin, and the fishing ban has been implemented from 1 March to 30 June every year [14]. Despite this, frequent utilization of hydraulic resources, thousands of years of agricultural development history, and high modern population density have caused a substantial disturbance of the freshwater fish ecosystem in the Pearl River Basin [37,38].

**Figure 1:**
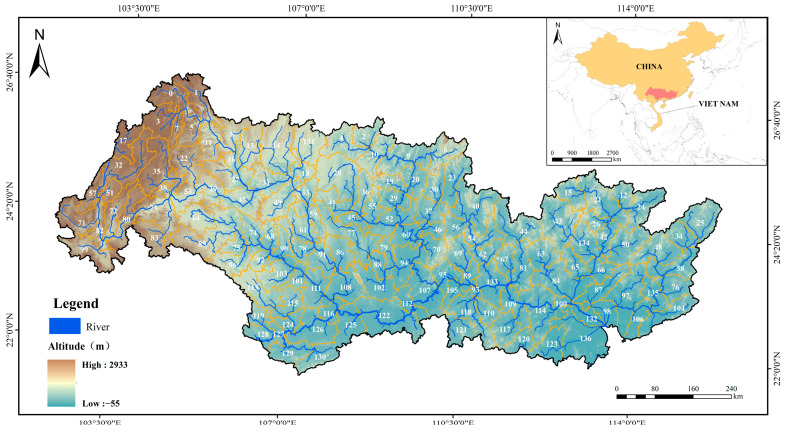
The sub-basin division map of the Pearl River Basin. The numbers (0~136) represent the identification numbers of different sub-basins, and the red area is the study area of this paper.

The sub-basins’ division was screened according to the following process: First, the “Near Analysis” toolbox in ArcGIS10.2 was used to correct the occurrence locations of all species to the water area. Next, a buffer was established for each occurrence point for all species [39,40]. Buffer distances for each occurrence point for each species were estimated based on fish dispersal capacity. Functional characteristics (i.e., full length and caudal fin aspect ratio) related to dispersal ability were extracted from the FISHMORPH database [41] and FishBase [42] for each species. These specific traits were selected as dispersal indicators because body size (full length) is strongly correlated with swimming endurance and active dispersal capacity in freshwater fish, while the caudal fin aspect ratio determines swimming efficiency and burst speed, which are critical for upstream migration and lateral movement across different habitat types [40]. The river grade at each occurrence point was also extracted from HydroRIVERS [43]. The buffer distance of each occurrence point was estimated with the R package ‘fishmove’ [40]. The estimated diffusion distance was used as the radius to construct a buffer for each occurrence point. Then, the buffer zone was superimposed with the sub-basins of level 7 (the average area was 3.3 × 10^3^ km^2^) in the HydroBASINS [43]. Level 7 was used because coverage at the point of occurrence was estimated to be lower at finer scales, namely levels 8–12, according to the compiled database. Moreover, based on estimates of dispersal capacity and existing studies, fish community composition varied considerably at higher levels (i.e., levels 1–7) [39,40]. However, larger sub-basins, such as level 6, may include more complex water body types, making it difficult to distinguish the effect of receiving water body types on the cumulative human pressure. For sub-basin clipping, we took the sub-basins where the occurrence point existed as the distribution range of the species, and those upstream or lower sub-basins of the occurrence point overlaps with the buffer zone were also listed as its distribution sub-basins. The ζ diversity of freshwater fish sub-basins (*n* = 137) of the Pearl River Basin was then assessed (Figure 1).

### 2.2. Fish Occurrence Records

A comprehensive review of scholarly sources was meticulously carried out across several repositories, encompassing Web of Science, Scopus, and CNKI, with an emphasis on monographs, digital repositories, such as FishBase and GBIF, alongside grey literature. Emphasis was placed on Chinese publications to encapsulate the full spectrum of native ichthyofauna in the Pearl River Basin, enhancing the breadth of the investigation. The initial inquiry was launched in October of the year prior to last (2022), with a subsequent update in the spring of the following year (May 2023). Adherence to the PRISMA guidelines ensured a structured and transparent selection process [44]. The resulting dataset underwent rigorous validation by two seasoned ichthyologists, with any dubious entries being expunged. This inquiry also gauged sample sufficiency across each sub-basin to pinpoint data-deficient regions and standardize inter-basin comparisons. Sub-basins with only one survey location or one species recorded were first removed, and sample coverage based on species richness (*q* = 0) was calculated on raw incidence data with extrapolation to two times the sample size. Only sub-basins with at least 90% coverage were retained for analyses, as this threshold ensures adequate representativeness of species communities while maintaining sufficient statistical power [27], yielding a final set of 14,308 with native fish occurrence records. Temporal observations within these basins spanned from 1984 to the present year. The study encompassed 579 fish taxa, of which 537 were native (both freshwater and diadromous), with the IUCN recognizing 200 as threatened (VU, EN, and CR) within the basin. Moreover, 78 taxa have been accorded national protection status under Classes I and II. The remaining taxa consisted of 35 non-native species to the Pearl River ecosystem and 7 species classified as extinct (EX) by IUCN assessment—these were consequently excluded from the species pool in the subsequent analyses. The prevalence of non-native species served as a surrogate for anthropogenic pressure, aiding in the examination of native biodiversity patterns.

In addition, this study collected fishery statistics data from the provinces (autonomous regions or municipalities directly under the Central Government) in the China Fishery Statistical Yearbook (1981–2022). According to the proportion of the Pearl River Basin in the waters of the provinces, the freshwater fishing output, fishery labor force, the number and engine capacity of fishing boats, the intensity of fishery supervision (measured by the number of workers and employees of fishery administration units and the number of fishery administration units), and the annual per capita income of fishermen in the Pearl River Basin were calculated. At the same time, the catch fishery and fish diversity in the Yangtze River Basin were compared with that in the Pearl River Basin, and the changes in the catch fishery in the Pearl River Basin in the past 40 years were analyzed. Based on the changes, we determined whether the Pearl River Basin has priority in the conservation of freshwater fish species among major river basins in China.

### 2.3. Variable Collection and Processing

In the pursuit of comprehending the spatial distribution of freshwater ichthyofauna, this investigation incorporated a multifaceted environmental data compendium. The environmental dataset included 19 environmental layers (raster data), including forest coverage, normalized vegetation index (NDVI), enhanced vegetation index (EVI), average light intensity, NPP vegetation primary productivity, evapotranspiration, and leaf area index. Human stress variables (GDP, fishing pressure, population density, mean fluctuation, road network density, water network density, arable area share, grassland area share, building area share, unused land area, and riparian disturbance (as a percentage of total land cover change within the 30 m riparian zone)), the number of large power stations, and 19 bioclimatic factors were extracted from Chelsa-climate (http://chelsa-climate.org, spatial resolution of 30 arc-seconds, accessed on 29 June 2023). Average annual fishing pressure data (t/km^2^) were obtained from the local fishery statistics yearbooks of each province and city for the last five years, weighted by the area of freshwater in each sub-basin per unit basin area. Prior to analysis, all environmental and climatic raster layers were standardized to a 1000 m resolution through cropping, resampling, and averaging processes, aligned within each sub-basin to ensure analytical precision. Moreover, spatial delineations of hydrological confines and geospatial attributes were assimilated, bolstering the predictive capacity of the model and facilitating a comparative assessment of these determinants against anthropogenic stressors, as proposed by Iacarella [27].

Recent research has elucidated that climatic gradients and elevational variance significantly influence beta diversity among freshwater fish across extensive spatial dimensions [45]. Ecological predictors, such as arboreal density, foliar biomass indices, and spectral vegetation signatures (NDVI and EVI), along with hydric flux and primary productivity metrics, have been validated as reliable proxies for forecasting ζ diversity in both flora and fauna [35,46]. In the context of the Pearl River Basin, anthropogenic disturbance metrics have demonstrated a robust correlation with species richness, echoing findings that underscore the pivotal role these stressors play in modulating species turnover within aquatic habitats [27,47]. Direct quantification of anthropogenic impacts on fish communities, such as those arising from increased fishing effort and fluvial habitat disruption, remains a formidable challenge in landscape ecology, particularly over expansive geographical scales [27]. Consequently, surrogate landscape indices of human disturbance are frequently employed as indicators of the cumulative effects on ichthyological communities and their habitats. These indices have proven instrumental in delineating patterns of fish diversity [27,35]. For this study, an evaluation of 19 anthropogenic pressure variables was conducted. Only those exhibiting weak correlation (|r| < 0.6) were retained for further analysis. The selected variables encompassed fishing pressure, GDP, surface temperature, leaf area index, proportion of arable land area, riparian disturbance, and number of power stations with generation capacity > 50 MW. Additionally, the prevalence of alien species was considered a distinct variable indicative of human-induced stress, given the Pearl River Basin’s status as a hotspot for ichthyological invasions, which significantly influences the distribution of endemic fish species [47,48]. Euclidean distances between sub-basins were computed using spatial coordinates to assess the influence of geographical separation on community heterogeneity. The extent of geographical dispersion was found to correlate with community divergence. Utilizing QGIS3.8, the area and centroid coordinates of each sub-basin were calculated, with subsequent logarithmic transformation to meet assumptions of normality. A principal component analysis (PCA) was then applied to 19 bioclimatic predictors to distill the environmental variables, considering their inherent collinearity (refer to Figure S1 for the correlation matrix). PCA facilitated a reduction in environmental predictors, thereby sharpening the focus on anthropogenic stressors. The first two principal components accounted for 88.7% of the variance, with the mean temperature of the warmest quarter (|r| = 0.97) and annual temperature range (|r| = 0.95) exhibiting high correlation. Consequently, these temperature metrics were utilized in the diversity analysis. Ultimately, the selected variables representing human pressure, environmental conditions, and geographical space were confirmed to be independent (|r| < 0.6), as this threshold ensured effective multicollinearity reduction while preserving ecological information content.

### 2.4. Sub-Basin Level Analyses

Elevational gradients have been identified as pivotal determinants of ζ diversity within lotic ichthyological communities [27]. To elucidate this relationship, our analysis stratified sub-basins into elevation-based cohorts, encompassing the entire spectrum of basins. Employing a k-means clustering approach, we delineated sub-basins into three to five clusters, achieving homogeneity in elevation without succumbing to model overfitting, as indicated by the high proportion of variance explained (91.4–96.6%; refer to Figure S2). The selection of an optimal cluster count was informed by the Ward’s minimum variance method, applied within a hierarchical clustering on principal components framework (‘FactoMineR’ package in R; [49]), which substantiated the superiority of a tripartite division. Consequently, three elevation-based clusters were constituted to streamline the complexity of downstream analyses. These clusters were demarcated into high (1458–2116 m, *n* = 19), intermediate (711–1302 m, *n* = 23), and low elevation (4–691 m, *n* = 95) bands, with each band’s human stress factors, environmental variables, and geospatial characteristics being evaluated within its respective altitudinal context (as depicted in Figure S3). Despite the notable difference in sample sizes across elevation bands, particularly the smaller number of high-elevation sub-basins (*n* = 19) compared to low-elevation sub-basins (*n* = 95), statistical validation through bootstrap resampling confirmed the robustness of our results, indicating that this sample size disparity did not significantly compromise the credibility of our findings.

Disaggregating ζ diversity, inclusive of its β-diversity component (ζ_2_), into facets of species turnover and nestedness offers critical insights for biodiversity management strategizing. Species turnover encapsulates the concept of distinct species assemblages between habitats, whereas nestedness implies that species-poor communities represent subsets of more diverse ones along a biotic gradient [50]. Understanding the proportional influence of these components on overall zeta diversity is instrumental for prioritizing conservation efforts, whether safeguarding ecologically unique locales or bolstering sites of pronounced local species richness to preserve regional biotic diversity [50,51]. The initial step involved quantifying total zeta diversity via the Sorensen index, subsequently partitioning it into components of species turnover (measured by Simpson’s index) and nestedness (as derived from the Sorensen–Simpson relationship), to appraise their relative significance both basin-wide and within specific elevational strata (‘adespatial’ package in R; [50,52]). The analytic continuum integrated both Sorensen and Simpson indices to encapsulate total ζ diversity and its turnover aspect. To elucidate the variance in ζ diversity of lotic fish fauna across the Pearl River Basin as a function of environmental and geographical heterogeneity among sub-basins, a multi-site generalized dissimilarity modeling approach was employed (‘zetadiv’ package in R; [53]). The characteristic decay of zeta diversity with ascending zeta orders offers insights into community assembly processes—whether stochasticity (exemplified by an exponential decay) or environmental filtering (indicated by a power-law relationship) predominates, with these dynamics potentially shifting across different orders [54,55]. Thus, the MS-GDM framework was harnessed to compute zeta diversity across various orders for all sub-basins and within each elevational tier. Commencing with the average zeta values for consecutive orders, we then applied linear regression fitting and power-law modeling to the logarithmically transformed data, selecting the most parsimonious model for the decline in zeta diversity with increasing order based on the Akaike information criterion (AIC).

In this study, ecological modeling was conducted utilizing Sorensen and Simpson diversity indices, taking into account a suite of 12 environmental predictors across the examined basins. For the Sorensen index, ζ diversity was normalized through the ratio of shared species across n sub-basins to the mean species richness within these sub-basins (ζ_n_/ζ_1_, with ζ_1_ representing mean species richness per sub-basin). Conversely, the Simpson index was normalized against the minimum species richness encountered in the sub-basins (ζ_n_/min(ζ_1_)) [56]. The multi-site generalized dissimilarity modeling (MS-GDM) employed generated non-linear, monotonic interpolations, specifically I-splines, normalized between 0 and 1 for each variable after adjustment (normalization achieved by subtracting the minimum and scaling to the range). Each I-spline curve delineated a cumulative impact metric for the sub-basins, aggregating all human pressure indices into a weighted sum, wherein the weights were informed by the non-linear impact of anthropogenic stress on ichthyofaunal compositional turnover [27]. These cumulative impact metrics served as indicators to pinpoint fish communities less influenced by human activities, potentially flagging areas of significant conservation interest, as well as those more heavily impacted, thus highlighting regions where restoration should be prioritized [23,57]. The apex of an I-spline curve signifies the overarching relative importance of a variable, while the slope variations within the curve indicate the range over which the variable most significantly impacts ζ diversity. To ensure robustness, each model iteration was conducted 30 times, with MS-GDM randomly sampling 1000 combinations of sub-basins for each iteration. This randomization allowed for consistent sub-basin sample sizes across both elevational clusters and zeta orders. Model performance was evaluated by the Pearson *r*, comparing observed to predicted values, to quantify the explanatory power of the MS-GDM [28,54,56].

The utility of the I-spline curve’s cumulative effect diminished when the synthesized zeta values across multiple sites exhibited minimal variability or when the model’s incorporated pressure variables failed to capture such variability, thus undermining the effective delineation of areas necessitating management intervention [27]. Within the lower-altitude sub-basins, the high explanatory power of the model variables and the pronounced fluctuation of zeta values between orders 2 and 3 (see Figure S4) guided the selection of Simpson diversity ζ_2_ for juxtaposition with paired Local Contribution to Beta Diversity (LCBD) indices. This approach facilitated a comparative analysis of anthropogenic impact with sub-basins’ compositional uniqueness to evaluate the appropriateness of these metrics for guiding management priorities. The distinctiveness of sub-basins’ composition within each elevational band was quantified using the Local Contributions to Beta Diversity based on Simpson’s diversity (utilizing the ‘adespatial’ package in R; [52]). Subsequently, for each sub-basin situated at lower elevations, cumulative effect estimates were juxtaposed with LCBD values to unravel the interplay between these two metrics. In this analysis, LCBD values were treated as the dependent variable, conforming to a normal distribution, while the cumulative effect served as the independent variable in a linear regression model to determine their relationship. All statistical procedures were conducted using R version 4.3.0, with analyses employing packages including fishmove, zetadiv, adespatial, FactoMineR, and base R functions. Independent sample *t*-tests were used to compare means between river basins after confirming normality (Shapiro–Wilk test) and homogeneity of variance (Levene’s test) assumptions.

## 3. Results

**Figure 2:**
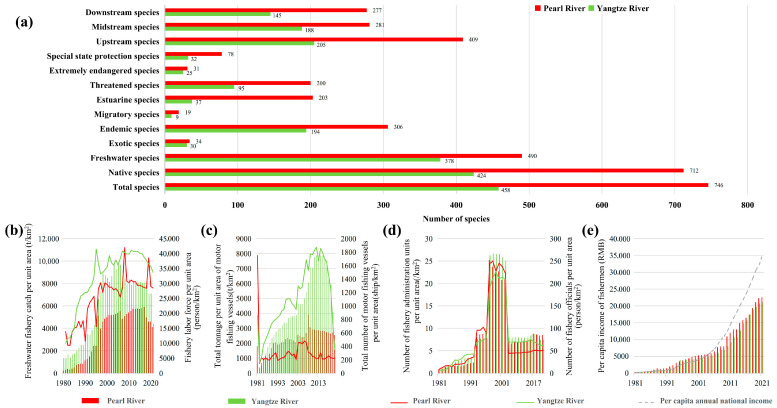
Comparison of freshwater fishery status in the Pearl River and Yangtze River Basin in the last 40 years. (**a**) The number of fish species in the basin. (**b**) Unit catch yield of freshwater fisheries and fishery labor force. (**c**) The number and tonnage of motor fishing vessels per unit. (**d**) The number of staff of fishery administration units and the number of fishery administration units. (**e**) Annual per capita income of fishermen and national per capita income. The legend on the left *Y*-axis is the polyline, and the legend on the right *Y*-axis is the column.

The number of fish and threatened species in the Pearl River Basin was twice that in the Yangtze River Basin (Figure 2a). Although the fishery labor force per unit (Figure 2b) and the number of motor fishing vessels per unit (Figure 2c) were much lower than those in the Yangtze River Basin, the fishery output per unit of the freshwater fishery (Figure 2a) and the intensity of fishery supervision (Figure 2d) had no significant difference from those in the Yangtze River Basin through the independent sample *t*-test (*p* > 0.05, Cohen’s d < 0.2, indicating negligible effect sizes). In addition, the annual per capita income of fishermen has been gradually lower than the national per capita income since the beginning of the 21st century (Figure 2e), which indicates that the freshwater fishery status of the Pearl River Basin and the Yangtze River Basin are also facing severe threats from fishing. Because of the diversity of freshwater fish and the particular freshwater environment, it is urgent to take further protective measures.

According to the classification of β diversity, the contribution of species turnover to the total β diversity was much greater than that of nestedness (turnover accounted for 78.32%, Sorensen = 0.36). High-altitude and middle-altitude species turnover accounted for 88.51% (Sorensen = 0.42) and 79.27% (Sorensen = 0.32), respectively, while low-altitude species turnover (47.21%) and nestedness (52.79%) contributed equally to β diversity (Sorensen = 0.31).

Analysis of zeta diversity metrics revealed a notable contraction in the number of shared species across sub-basins to fewer than 60 when the comparison extended beyond 2 sub-basins (zeta order ≥ 3), encompassing the entire basin, as well as within mid- and high-elevation sub-basin groupings (refer to Supplementary Table S1 and Figure S4). In contrast, low-elevation sub-basins exhibited a higher degree of species overlap, with a minimum of 60 species commonly found in comparisons involving up to 14 sub-basins. Furthermore, the observed patterns of zeta diversity reduction across the gradient from high to low elevations suggested an increasing influence of environmental filtering on the structuring of aquatic communities (as shown in Table 1). Across the entire basin and within individual elevational basins, both exponential and power-law models were fitted to the data, with the power-law model demonstrating superior fit. This pattern indicates that community assembly was predominantly influenced by environmental filtering, as evidenced consistently across the first three zeta orders.

Results from the model showed an increase in the percentage variance of variable explanations from high- to low-altitude sub-basins, consistent with an increase in filtering in low-altitude environments, as shown by the decline in zeta diversity (Table 1 and Table 2). The variance rate of model interpretation for low-altitude sub-basins increased with the increase in the number of basins at orders 2–4, Sorensen and Simpson diversity begin to decline at orders 6 and 5, respectively, and then the sets became more random (only the results of orders 2–4 are provided for the sake of simplicity in the subsequent analysis). The variance explained by Sorensen was also generally higher than Simpson’s diversity (Table 2). The contrasting patterns between exponential and power-law fits across elevation gradients (Table 1) combined with variable explanatory capacity (Table 2) highlight the differential sensitivity of Sorensen and Simpson indices. The higher explanatory power observed for Sorensen diversity (e.g., 65.40% for all sub-basins vs. 62.19% for Simpson) reflects its greater sensitivity to species richness differences, making it more effective for detecting comprehensive biodiversity patterns across environmental gradients. Conversely, Simpson diversity’s relatively stable performance across elevation bands (ranging from 50.98% to 56.16%) indicates its reduced sensitivity to richness variations, allowing it to better capture true compositional turnover patterns for targeted restoration strategies in degraded areas.

**Figure 3:**
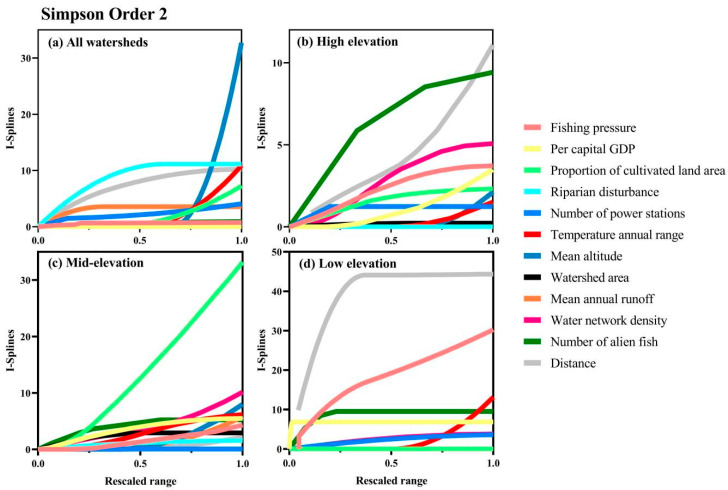
Depicts the average I-splines derived from MS-GDM analyses to illustrate the influence of anthropogenic pressures, environmental conditions, and geospatial attributes on species turnover, as assessed by Simpson zeta diversity among paired sub-basins (‘order 2’). This is presented for (**a**) the collective set of sub-basins, as well as segregated by (**b**) high-, (**c**) medium-, and (**d**) low-elevation sub-basins. The apex of the I-spline curve signifies the relative significance of each variable in elucidating zeta diversity, while the slope’s fluctuation reflects the changing pace of community dissimilarity across the variable gradient. The x-axes represent the normalized range of variables from 0 to 1. Notably, elevation was exclusively incorporated in the models for the comprehensive sub-basins analysis (**a**).

**Figure 4:**
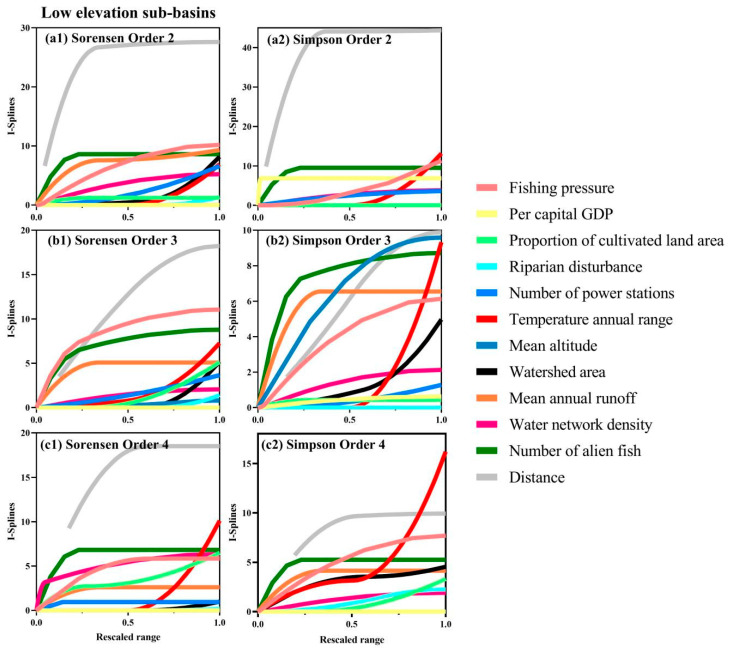
This figure illustrates the mean I-splines generated through MS-GDM, which quantify the impacts of anthropogenic, environmental, and geospatial factors on (**1**) the aggregate zeta diversity (‘Sorensen’) and (**2**) the species turnover (‘Simpson’) within low-elevation sub-basins across zeta orders (**a**) 2, (**b**) 3, and (**c**) 4. The peak value of each I-spline curve denotes the comparative contribution of the variable to the explanation of zeta diversity, while alterations in the slope’s trajectory reveal shifts in the rate of community dissimilarity across the variable’s spectrum. Pronounced I-splines for ‘Sorensen’ without corresponding ‘Simpson’ effects highlight influences on the nestedness aspect of zeta diversity. In contrast, I-splines associated with ‘Simpson’ delineate impacts on the turnover facet. The x-axes present the variables normalized from 0 to 1.

**Figure 5:**
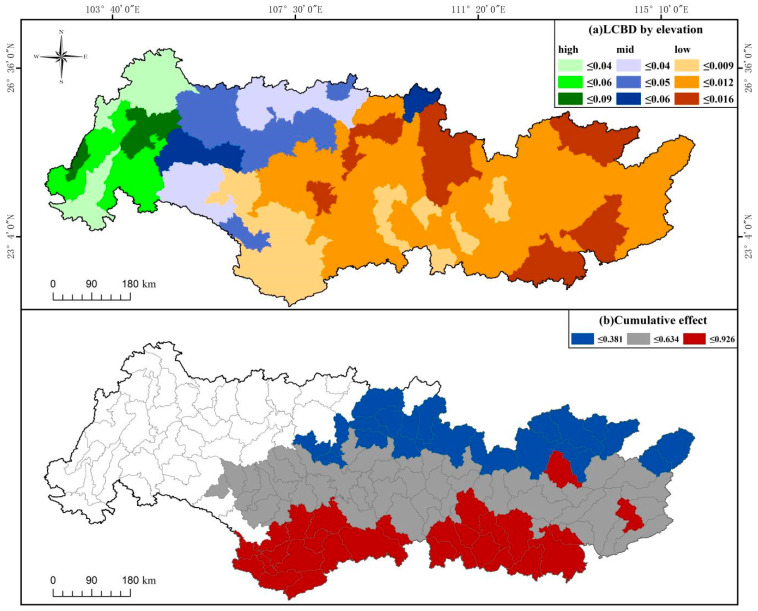
Presents the prioritization strategies for freshwater biodiversity conservation within the Peal River Basin, pinpointed through (**a**) the distinctiveness of ichthyofaunal assemblages, evaluated via Local Contributions to Beta Diversity (LCBD) for Simpson diversity across varying elevations—high (depicted in green), medium (blue), and low (orange)—and (**b**) the aggregated impact of anthropogenic stressors on sub-basins at lower elevations.

**Figure 6:**
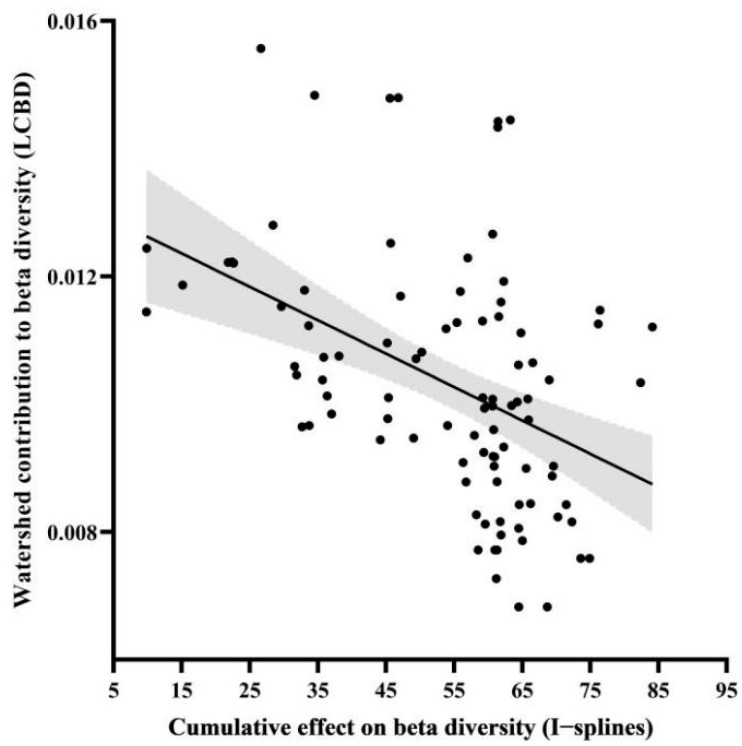
Delineates the inverse relationship between the distinctiveness of fish communities, quantified by Local Contributions to Beta Diversity (LCBD) for Simpson diversity, and the compounded anthropogenic disturbances within low-elevation sub-basins. These cumulative effects were computed as the aggregate of non-linear reactions of ichthyofaunal community turnover, ascertained through I-splines from multi-site generalized dissimilarity models. The graph includes shading that represents 95% confidence intervals obtained from a linear regression analysis (*p* < 0.001).

Mean I-splines of MS-GDMs were shown for Simpson diversity for all sub-basin groups (Figure 3 and Figure 4), as well as for Sorensen diversity for low-elevation watersheds (Figure 4), as beta diversity partitioning indicated an equivalent influence of turnover and nestedness only at low elevations. Among the components and orders of zeta diversity, climate factor, human pressure, and geospatial variables all ranked at the top of the maximum I-spline value, indicating that the above variables had relatively important influences on zeta diversity (Figure 3 and Figure 4). For all sub-basins, the largest driver of species turnover was the mean altitude, followed by riparian disturbance, geographic distance, annual temperature range, and proportion of cultivated land area. In contrast, the fishing pressure contributed minimally to the turnover component of zeta diversity (Figure 3a). In high-elevation sub-basins, geographical distance and the number of alien fish were the main drivers of turnover, which had a greater effect on composition, mainly when rescaled variables were low (<0.5), followed by water network density and fishing pressure at medium–high values (Figure 3b). The proportion of cultivated land area and water network density greatly affected the middle-elevation sub-basins (Figure 3c). In addition to geographical distance, the most significant driving factors in the low-altitude sub-basins were the fishing pressure, annual temperature range, and the number of alien fish (Figure 3d). The total zeta diversity at low altitudes varied greatly among different basins for orders 2–4, such as the fishing pressure, geographical distance, number of alien fish, annual temperature range, and mean annual runoff (Figure 4a,c). These variables were also crucial for the turnover of low-altitude basins. This suggests that the fishing pressure, geographical distance, the number of alien fish, annual temperature range, and the mean annual runoff mainly influenced the turnover component of the total zeta diversity (Figure 4a,c). The LCBD, composed of unique species, quantified important sub-basins within each elevation (high, middle, and low) of the Pearl River Basin (Figure 5). In addition, the lower cumulative environmental effect measured the higher LCBD and the fewer unique communities of the basin with a greater cumulative effect on beta diversity (*r* = 0.88, *t* = −5.058, *p* < 0.01; Figure 5 and Figure 6).

## 4. Discussion

The Pearl River Basin faces urgent conservation challenges, as fishing incomes remain below national averages, while per-unit freshwater fishery harvests exceed those of the Yangtze River, where a decade-long moratorium has been implemented. Fishermen’s dependency on freshwater fish for livelihoods drives overexploitation, with nearly all aquatic species targeted commercially after the brief four-month fishing hiatus, directly impacting fish abundance and diversity [58]. Despite harboring China’s highest freshwater fish species richness and endemism, the Pearl River Basin receives insufficient conservation attention compared to the Yangtze River, partly due to the need for conservation strategies informed by both large-scale patterns and localized distributions [19,59].

This study addressed critical gaps in evaluating freshwater fish responses to complex anthropogenic stressors by establishing a novel linkage between beta diversity and cumulative impacts. Our enhanced methodology incorporated qualitative vulnerability weights derived from species’ responses to stressors, using zeta diversity and multi-site generalized dissimilarity modeling (MS-GDM) to highlight relative stressor importance. By connecting cumulative impact assessments to species assemblage distinctiveness across sub-basins (LCBD), we identified priority conservation areas. This integrated approach provided actionable insights for fishery managers by strategically focusing conservation and management initiatives based on regional distinctions, species-specific responses, and anthropogenic pressures [24,27].

Within the Pearl River Basin, freshwater fish zeta diversity showed disparate responses to anthropogenic stressors, including fishing pressure, riparian disturbance, alien fish numbers, cultivated land proportion, and water network density. Many significant shifts occurred at lower impact thresholds, plateauing beyond higher levels. Fish community composition changed markedly with minimal urban and agricultural development, while species richness showed reduced sensitivity [47]. Therefore, biodiversity assessments should emphasize species turnover over richness, and cumulative effect evaluations must highlight non-linear diversity–stress dynamics [27]. Concentrating human activities within smaller sub-basins appears more effective than dispersing low-impact activities throughout entire regions. The MS-GDM’s explanatory capacity aligns with other ecosystem studies [27,35], with fishing pressure and GDP differentials significantly influencing species turnover at lower elevations, while having minimal effect at higher elevations.

This investigation compiled ichthyofaunal distribution, environmental variables, and anthropogenic pressures within Pearl River Basin sub-basins. The inverse relationship between cumulative anthropogenic stress (MS-GDM) and fish species composition distinctiveness (LCBD values) supported these metrics for conservation prioritization. Areas with low cumulative effects and high LCBD scores are prime candidates for Kunming-Montreal Global Biodiversity Framework objectives [23,27]. Karstic middle-to-high-elevation sub-basins harbor numerous endangered cave-dwelling endemic species requiring urgent protection due to inadequate survey data [60]. Conversely, sub-basins with elevated cumulative impacts and reduced LCBD may undergo biotic homogenization, necessitating targeted restoration. The Pearl River Delta contains 131 rare or absent aquatic species (one-third of total ichthyofauna), including *Acipenser sinensis* and *Tanichthys albonubes*. Overfishing, compromised water quality, and habitat conversion for economic development severely threaten delta fish populations [37,38].

Historically, the Pearl River and Yangtze River basins shared analogous environmental challenges, where brief annual moratoria on fishing failed to yield the anticipated conservation outcomes due to pervasive overexploitation of fisheries, aquatic contamination, coastal land reclamation, and rampant sand extraction activities [14,16]. The absence of a ‘fishing flood’ phenomenon in the Pearl River Basin, coupled with a persistent downtrend in biodiversity metrics, underscores the stark reality of an ecosystem under siege [61]. A ‘fishing flood’ refers to the seasonal surge in fish catches that typically follows spawning periods when restrictions are lifted, indicating healthy fish population recovery. Its absence signals the failure of current short-term fishing bans to restore fish stocks. Temporal or localized prohibitions on fishing are insufficient to stem the ecological crisis in the basin, given the resumption of unregulated harvests post-spawning season and the confluence of multiple, intensifying anthropogenic stressors [14,58]. The integration of Local Contribution to Beta Diversity (LCBD) indices with assessments of cumulative impacts offered considerable insight for prioritizing biodiversity conservation and identifying requisite rehabilitative measures. A blanket fishing prohibition across the entire Pearl River Basin is not advocated, as the fishing pressure minimally impacted the turnover component of zeta diversity within all sub-basins. Moreover, alien fish species significantly affected the zeta diversity of fish assemblages across various elevations. Consequently, fishing restrictions aimed at protecting native species might inadvertently provide reproductive opportunities for these invasive groups [14,48].

Therefore, this study proposes the following:

(i) Effective restoration of aquatic life and fisheries may be achieved through the strategic adjustment of human-induced pressures and stressors, in conjunction with stringent fishery regulations [14]. This approach necessitates the establishment of an extensive monitoring framework for the Pearl River ecosystem, the enhancement of current fishing restrictions in both duration and coverage, rigorous enforcement of said regulations, and the initiation of large-scale ecological rehabilitation initiatives. Such measures are crucial for the revival of the Pearl River Basin, home to China’s richest freshwater fish diversity and fisheries. This model is also pertinent to developing regions in Africa, Southeast Asia, and South America, where similar patterns of economic advancement and population growth are accompanied by the ecological deterioration of aquatic habitats (Chen et al., 2020) [14].
(ii) To promote the recovery and replenishment of aquatic species, a 10-year moratorium on fishing is proposed to be implemented in the waters of Guangdong–Hong Kong–Macao (GH-MO) Greater Bay Area (Pearl River Delta) in view of the high cumulative effects of human pressures (e.g., fishing pressure) and species rarity in the low-elevation Pearl River Delta. This conservation strategy, which is inextricably linked to the National Development Agenda, aims to raise the ecological quality and governance of the Guangdong–Hong Kong–Macao Greater Bay Area to global standards and implement the most stringent environmental protection protocols.
(iii) A compensatory framework is suggested for the fishing prohibition in the Guangdong–Hong Kong–Macao Greater Bay Area, which would steer the Pearl River Basin Delta’s fishing community toward alternative livelihoods, expediting their shift from fishing to other forms of production. This transition should be supported by comprehensive policies addressing employment and social security for the fishermen transitioning out of the industry.
(iv) Special consideration is warranted for the conservation of aquatic ecosystems, including groundwater, within the karst regions of the Pearl River Basin. Intensified monitoring and protection of freshwater fish biodiversity in these areas are imperative. A substantial body of fundamental research is required to ascertain the endangered status and categorize the risk levels of fish species inhabiting these karst environments.
(v) By implementing successful fisheries regulation and mitigating anthropogenic impacts, China can enhance freshwater management while leading by example for developing nations. Future management should integrate advanced technologies, including remote sensing, environmental DNA (eDNA) monitoring, and real-time water quality sensors, for dynamic, adaptive management. Satellite-based habitat monitoring combined with machine learning algorithms can provide early warning systems for biodiversity loss. Establishing international collaborative platforms for sharing monitoring data, management protocols, and technological innovations will enhance the global freshwater biodiversity conservation capacity, representing next-generation conservation strategies that move beyond static protection toward responsive ecosystem management.

## 5. Conclusions

This study reveals fish zeta diversity responses to human pressures in the Pearl River Basin, showing that cumulative pressure effects in low-elevation areas significantly exceed those in mid- to high-elevation regions and negatively correlate with community uniqueness. We recommend implementing long-term fishing bans in high-pressure areas like the Pearl River Delta while strengthening protection in mid- to high-elevation watersheds. Integrating zeta diversity analysis with cumulative effects assessment provides a novel framework for watershed ecosystem management and freshwater fish conservation, offering important guidance for sustainable fisheries management in developing countries.

## Figures and Tables

**Table 1:** The fitting of zeta diversity declines using exponential and power-law models across ascending zeta orders for both the collective sub-basins and those categorized by elevation. The model providing a superior fit was identified by an Akaike information criterion (AIC) value that was at least 2 units lower (indicated in bold). Instances where a shift in the fitting model occurred across different orders are highlighted. An exponential fit is suggestive of stochastic processes governing community assembly, while a power-law fit implies a deterministic community structuring influenced by environmental filtering mechanisms.

Sub-Basins Group	Order of Zeta	Exponential Coefficient			Power-Law Coefficient		
		*b*	*p*	AIC	*c*	*p*	AIC
All	1–3	−0.11	0.15	−8.16	−0.49	0.06	**−13.98**
High	1–3	−0.08	0.22	−8.66	−0.34	0.11	**−12.44**
	1–4	−0.05	0.09	−11.18	−0.29	0.03	**−15.90**
Mid	1–3	−0.11	0.13	−9.22	−0.47	0.04	**−16.98**
Low	1–3	−0.09	0.16	−9.31	−0.38	0.06	−14.71
	1–4	−0.07	0.06	−11.65	−0.35	0.01	**−18.49**

**Table 2:** The proportion of variance elucidated (as indicated by the squared Pearson correlation coefficient, r), attributed to anthropogenic pressures, environmental factors, and geospatial determinants, as computed by MS-GDM. This encompasses the comprehensive zeta diversity (‘Sorensen’) and species turnover dynamics (‘Simpson’) for the second zeta order across all sub-basin clusters, as well as for ascending orders within low-elevation sub-basin groups. The reported values represent the mean ±1 standard deviation derived from 30 iterations of the model.

Sub-Basin Groups	Order of Zeta	Sorensen, *r*	Simpson, *r*
All	2	65.40% ± 5.95%	62.19% ± 6.67%
High	2	57.18% ± 8.04%	56.16% ± 5.14%
Mid	2	55.87% ± 5.25%	50.98% ± 5.61%
Low	2	55.56% ± 9.64%	54.79% ± 7.36%
	3	63.19% ± 6.63%	63.14% ± 6.12%
	4	66.99% ± 5.99%	65.61% ± 9.49%
	5	68.18% ± 7.86%	64.86% ± 6.62%
	6	67.17% ± 5.97%	63.39% ± 5.96%

## Data Availability

Data will be available upon request.

## Supplementary Materials

The following supporting information can be downloaded at: https://www.mdpi.com/article/10.3390/biology14070796/s1, Figure S1. Correlation plot for all human pressure and bioclimatic (CHELSA01-19) variables; Figure S2. K-means within cluster sum of squares across different numbers of cluster groups based on elevation; Figure S3. Range of values for human pressure, environmental, and geospatial variables and across watershed elevations as grouped by high, mid-, and low elevation clusters; Figure S4. Zeta decline for high, mid-, and low elevation watersheds shows the rate at which the number of shared species (‘zeta values’) decreases as more watersheds are compared at once (‘zeta order’); Table S1. Zeta values (number of shared species) for increasing orders of zeta (number of watersheds compared at once).
