# Supplementary material for: Responses of Fish Zeta Diversity (ζ) to Human Pressure and Cumulative Effects: A Feasibility Study of Fishing Ban Measures in the Pearl River Basin, China
Source: Biology (Basel). 2025 Jun 30;14(7):796. doi: 10.3390/biology14070796 (PMC12292702; doi:10.3390/biology14070796)
Supplement: Supplementary file 1 [file biology-14-00796-s001.zip › biology-3725491-supplementary.pdf]

Supporting Information. Preliminary results supporting decisions made for the fish zeta diversity analysis.

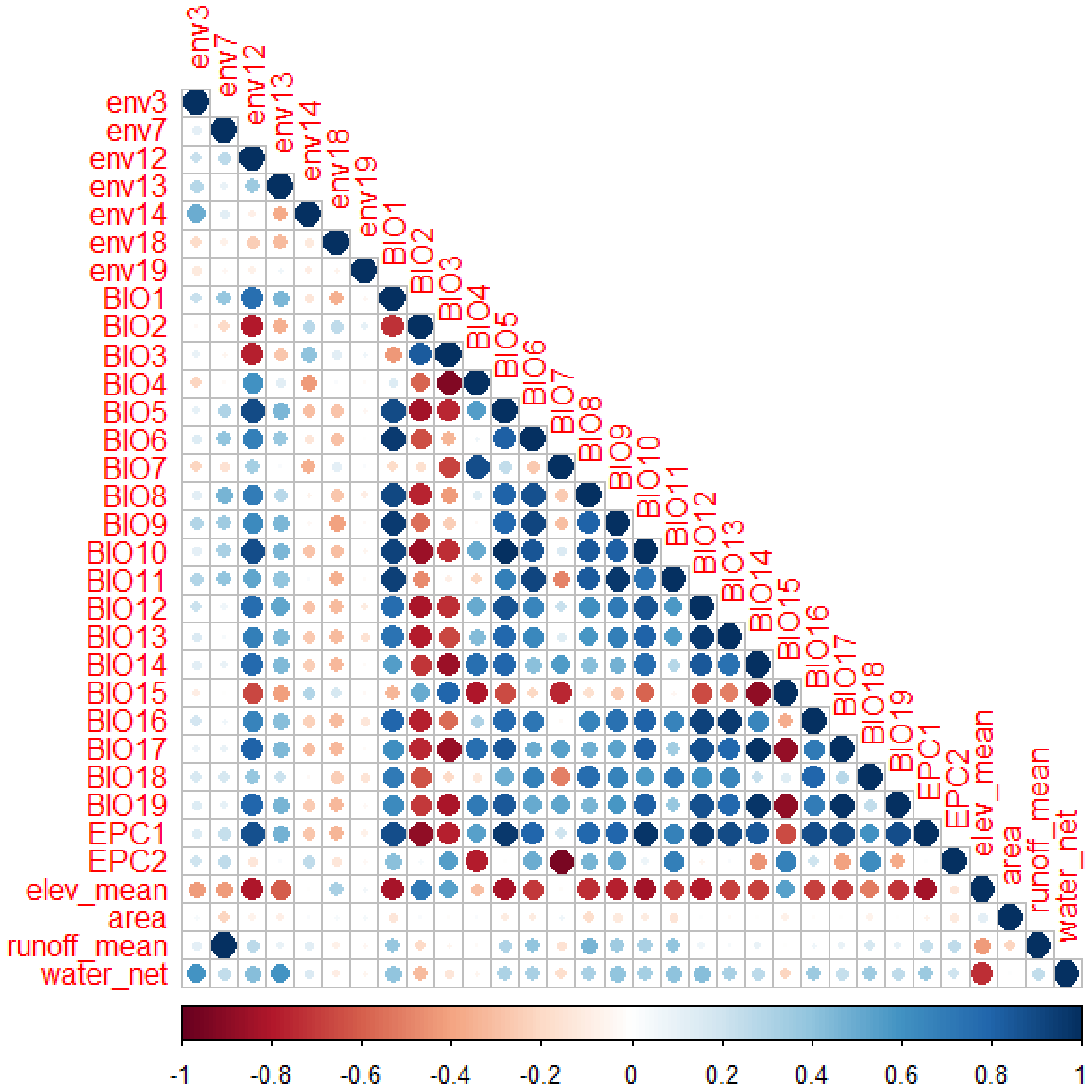

Figure S1. A heatmap of correlations among all human stress variables, 19 bioclimatic variables, and the first two principal component axes: All human stress variables include: average light intensity (env3); GDP (env7); proportion of arable land area (env14); riparian disturbance (env18); number of power stations with generation capacity >50MW (env19); mean elevation (elev_mean); watershed area (area); mean runoff (runoff mean); water network density (water_net); alien fish species (alien_fish); the first two principal components: EPC1 and EPC2; bioclimatic variables include: mean annual temperature (BIO 1); mean diurnal range (BIO 2); isothermality (BIO 3); temperature seasonality (BIO 4); maximum temperature of the warmest month (BIO 5); minimum temperature of the coldest month (BIO 6); annual temperature range (BIO 7); mean temperature of wettest quarter (BIO 8); mean temperature of driest quarter (BIO 9); mean temperature of warmest quarter (BIO 10); mean temperature of coldest quarter (BIO 11); annual precipitation (BIO 12); precipitation of wettest month (BIO 13); precipitation of driest month (BIO 14); precipitation seasonality (BIO 15); precipitation of wettest quarter (BIO 16); precipitation of driest quarter (BIO 17); precipitation of warmest quarter (BIO 18); precipitation of coldest quarter (BIO 19).

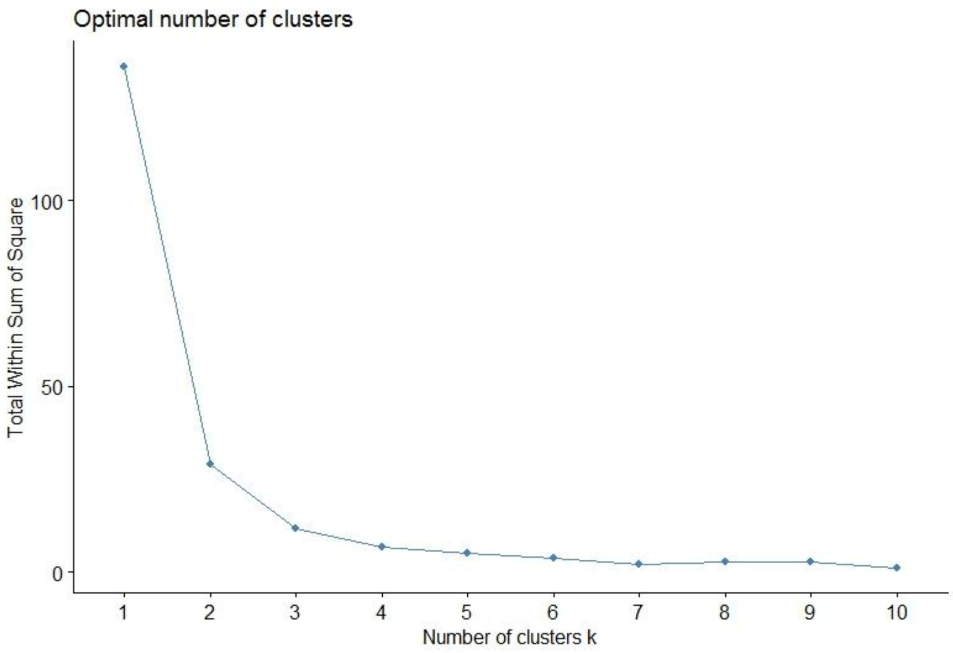

Figure S2. K-means within cluster sum of squares across different numbers of cluster groups based on elevation. The within cluster sum of squares indicates how dissimilar sub-basins are within a group.

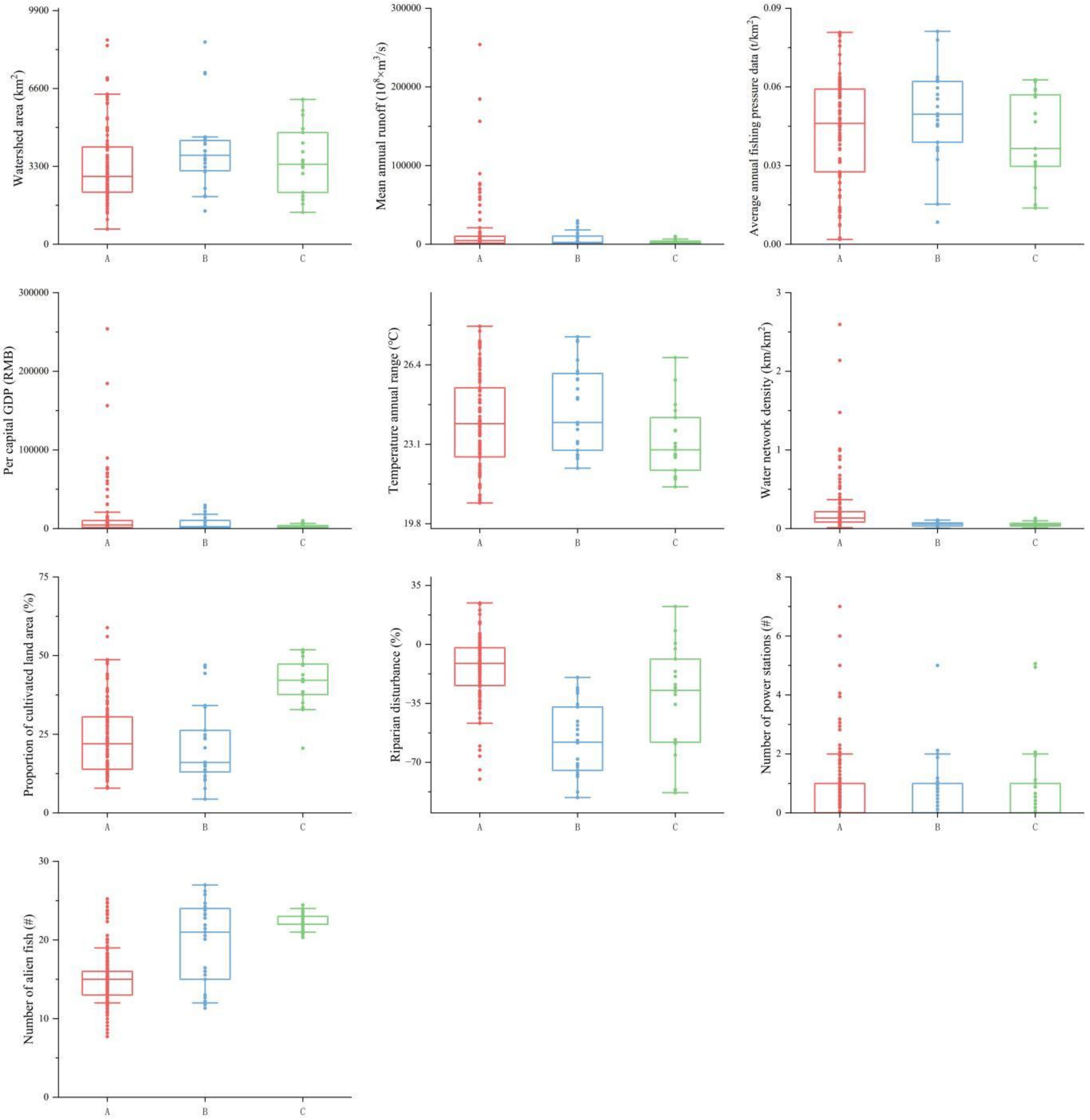

Figure S3. Range of values for human pressure, environmental, and geospatial variables and across sub-basin elevations as grouped by high, mid-, and low elevation clusters. The upper and lower edge lines of the box represent the upper and lower quartiles of the data, the horizontal line in the box represents the median of the data, and the upper and lower horizontal lines in the box represent the maximum and minimum values of non-departure data.

Table S1. Zeta values (number of shared species) for increasing orders of zeta (number of watersheds compared at once). Values are for all watersheds included in the analysis, and for high, mid-, and low elevation sub-basin groups.

**Order of zeta:**	1	5	10	15	20	21	22
Zeta values: All	104.44	53.11	45.85	43.61	42.76	42.66	42.57
**Order of zeta:**	1	5	6	7	8	-	-
Zeta values: High	73.11	48.41	47.9	47.58	47.37	-	-
**Order of zeta:**	1	5	8	9	10	11	-
Zeta values: Mid	93.17	48.34	45.35	44.95	44.67	44.47	-
**Order of zeta:**	1	5	10	13	14	15	16
Zeta values: Low	113.43	67.82	62.43	61.23	60.96	60.72	60.52

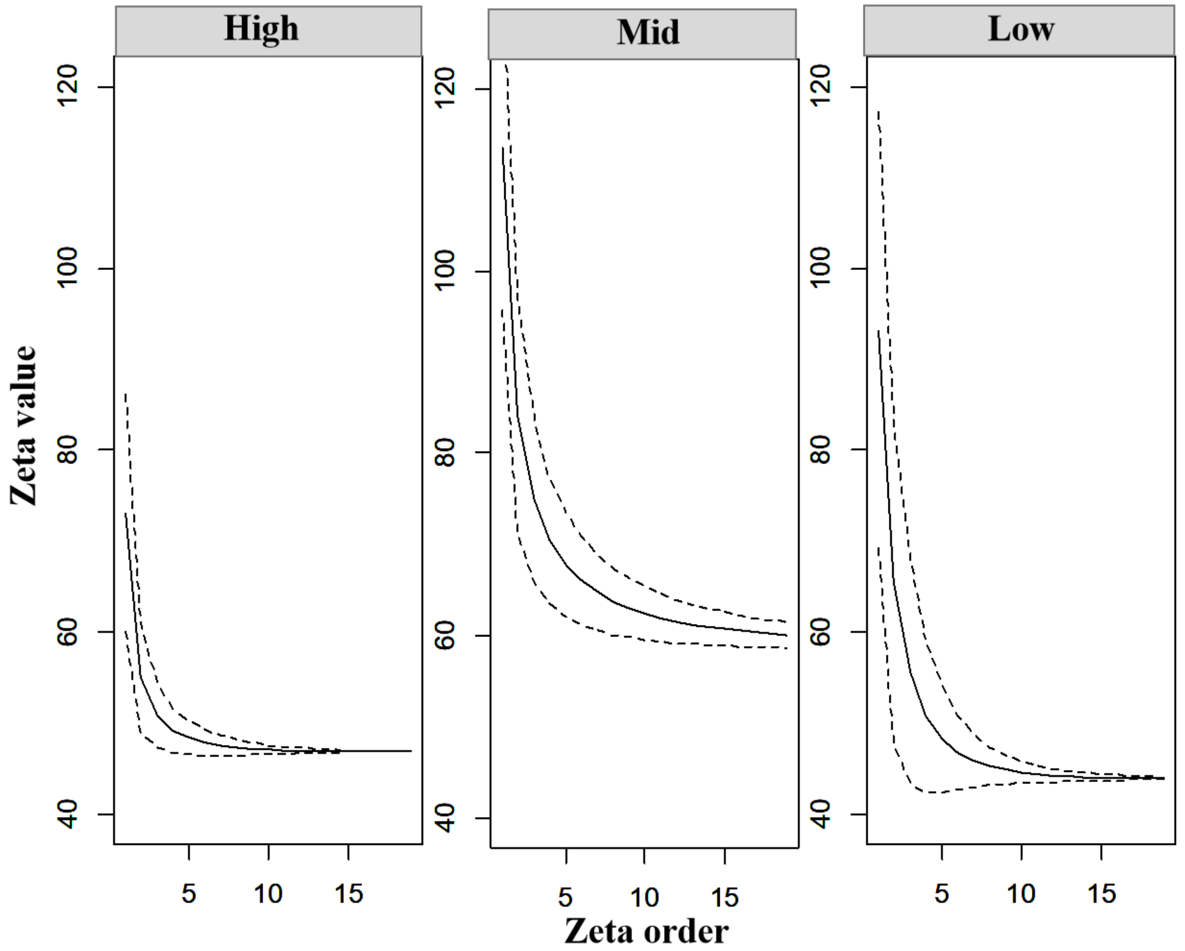

Figure S4. Zeta decline for high, mid-, and low elevation sub-basins shows the rate at which the number of shared species ('zeta values') decreases as more watersheds are compared at once ('zeta order'). Lines are mean ± 1 SD.
